# Undesired nexus poor health status of child under-five: A case study of Pakistan

**DOI:** 10.1371/journal.pone.0323845

**Published:** 2025-05-30

**Authors:** Urva Zainab, Mohsin Abbas, Amena Urooj, Mahwish Rabia, Huaping Sun, Muhammad Ahmed Shehzad

**Affiliations:** 1 PIDE School of Economics, Pakistan Institute of Development Economics, Islamabad, Pakistan; 2 School of Economics and Management, University of Science and Technology Beijing, Beijing, China; 3 Department of Statistics, Bahauddin Zakariya University, Multan, Pakistan; 4 Department of Statistics, Government College Women University Sialkot, Sialkot, Pakistan; Jahangirnagar University, BANGLADESH

## Abstract

Childhood morbidity and mortality are key indicators of human development, particularly reflecting poor health conditions in children. In Pakistan, child mortality remains a serious problem despite efforts to reduce it. One factor that may be associated with child mortality is an undesired pregnancy, whether unwanted (the parents did not want more children) or mistimed (the pregnancy occurred earlier than desired). Unwanted pregnancies and births are psychological factors that negatively impact children’s nutritional health. The main objective of the study is to measure the impact of mothers’ aspired status on child mortality and morbidity in Pakistan. We limited our analysis to children under 5 before the survey and used Pakistan demographic health survey conducted in 2017-2018, a national representative cross-sectional survey. We were able to predict the unwanted state (excess in boys, girls, both, and parity) by subtracting the ideal number of children from total live births. Morbidity (fever, diarrhea, cough, acute respiration infection, and Short rapid breathing), nutritional status, and mortality were also evaluated. We perform machine learning techniques such as random forest (RF) and neural network (NN) in the analysis of the data. The findings revealed that the overall percentage of the undesired child was 8%, 4%, 15%, and 27% for boys, girls, parity, and dual excess, respectively. Finally, we perform multivariate analysis following the principal component analysis (PCA) to study the relationship between variables. All the variables were associated with the unwanted child. Child morbidity, fever, and cough were higher among the undesired children. We found evidence that undesired children have acute respiration infection and that an unwanted child has a significant impact on childhood diseases. The ratio of child mortality was lower for boys but higher for girls.

## Introduction

Unwanted pregnancies—those that are mistimed or entirely undesired—pose a significant global public health challenge, contributing to adverse maternal and neonatal outcomes as well as increased social and economic burdens. Globally, about 38% of the 210 million annual pregnancies are unwanted, with 22% ending in abortion [1]. In Pakistan, a hospital-based cross-sectional survey reported that 38.2% of pregnancies were unwanted [2], with roughly 18% resulting in unwanted births, placing considerable strain on healthcare systems; [3].

Unintended pregnancies are a major public health concern, particularly in low- and middle-income countries, [4]. According to the Pakistan Demographic and Health Survey (PDHS) 2017-18, the prevalence of unintended pregnancies increased by 1%, reaching 25%; [5]. Reported estimates of unintended pregnancies in Pakistan vary between 16% and 46%, see [6], and [7] for more details. The PDHS surveys of 2006 and 2013 estimated the prevalence at 16% and 24%, respectively; [8]. These estimates were based on a single question with a dichotomous response regarding whether the pregnancy was mistimed or unwanted at the time of conception. Another study estimated the prevalence at 46%, using an indirect modeling approach based on induced abortion rates; [9].

Unintended pregnancies are a major cause of abortions, with additional contributing factors such as financial hardship, maternal health risks, and relationship problems [10–12]. These pregnancies are most prevalent in developing countries, largely due to low education levels, limited awareness, and restricted access to contraception. In such settings, unwanted pregnancies have profound health, social, and economic consequences. They significantly increase the risk of maternal, neonatal, infant, and child mortality, see [13–16], and the references cited therein. Research by [17] shows that children from unwanted pregnancies are more likely to suffer from illnesses and are three times more likely to experience stunted growth. Furthermore, [18] found that in three out of five countries studied, exceeding the desired family size was associated with higher rates of infant and child mortality.

Reducing unwanted childbearing is crucial for improving child health and survival, as it is closely linked to poor health outcomes in children [19,20]. In 2021, Pakistan reported an under-five mortality rate of 63.3 deaths per 1,000 live births, according to the World Data Atlas [21]. The leading causes of child mortality in underdeveloped countries, including Pakistan, are pneumonia, diarrhea, birth complications, malnutrition, malaria, and neonatal sepsis [22,23]. Despite global progress in child survival, approximately 15,000 children under five still die each day, with 90% of these deaths occurring in low-income countries [24]. Malnourished children are particularly at risk, facing higher mortality from common illnesses such as malaria, pneumonia, and diarrhea [20,25]. Nutritional deficiencies—manifesting as stunting, wasting, and underweight—are estimated to contribute to about 45% of deaths in children under five [19,21,24].

Among South Asian countries, Pakistan has the highest infant mortality rate. Investments in infant and child health often involve complex decisions regarding household resource allocation, where a mother’s preferences for family size and gender composition can play a significant role, see [26–28]. A study by [9] found that of approximately 9 million pregnancies in Pakistan in 2012, 4.2 million (46%) were unintended. Of these, 54% ended in induced abortions, while 34% resulted in an estimated 1.4 million unplanned births, imposing substantial economic, social, and health burdens on families, particularly mothers.

Pakistan has a low contraceptive prevalence rate (35.4%) and a high unmet need for family planning (FP, 20.1%), contributing to a fertility rate of 3.8 births per woman and a significant number of unintended pregnancies. The country records approximately 2.25 million abortions annually, with 50 abortions per 1,000 women aged 15–49. Due to abortion’s illegal status, many procedures occur in unsafe conditions, leading to complications. In 2012 alone, over 62,000 women required medical treatment for abortion-related complications [8,9,29].

Research highlights the widespread occurrence of induced abortions in Pakistan. [30] estimated that 890,000 abortions occur each year, with a rate of 29 per 1,000 women aged 15–49. Abortions account for one in every seven pregnancies, with higher rates in provinces where contraceptive use is lower and unintended pregnancies are more frequent. Supporting this, [31] found that 82% of abortions were due to unintended pregnancies, a trend also observed in Karachi by [32]. These studies collectively emphasize the strong link between unintended pregnancies and the high incidence of abortions in Pakistan.

While unintended pregnancy rates have declined in high-income countries[33], they remain significantly higher in low- and middle-income nations [34]. In Asia alone, approximately 53.8 million unintended pregnancies occur annually, with 5.4% of women aged 15–44 experiencing unintended pregnancies between 2010 and 2014 [35].

Although abortion is prevalent in Pakistan, a comparative analysis with neighboring countries provides valuable context. Pakistan’s abortion rate is estimated at 29 per 1,000 women aged 15–49 [30], while India reports a significantly higher rate of 47 per 1,000, reflecting better access to FP and safe abortion services [36]. In contrast, Bangladesh has a lower abortion rate but relies more on menstrual regulation due to restrictive abortion laws [37]. Pakistan’s restrictive abortion laws and limited access to contraception contribute to unsafe abortion practices, leading to serious maternal health risks. In 2012 alone, 62,000 women required medical treatment for complications from unsafe abortions [30]. These challenges highlight Pakistan’s need for stronger FP programs, as seen in its neighboring countries, to mitigate the impact of unintended pregnancies and unsafe abortions [36,38].

Numerous studies have sought to predict the health outcomes of unwanted children by accounting for key socioeconomic factors such as under-five child survival, morbidity, treatment access, and nutritional status. Major predictors of child health include maternal age, child sex, birth order, household size, wealth index, parental education, maternal nutrition, healthcare utilization, region, religion, and place of residence. However, Pakistan has yet to conduct a comprehensive analysis of child undesirability similar to [39], which was undertaken in Bangladesh. Research on newborn survival in Pakistan remains limited, lacking a strong conceptual framework and incorporating only a narrow range of influencing factors.

Machine learning (ML) techniques are widely used in statistics for both inference and prediction. In healthcare, supervised and unsupervised ML models aid in diagnosing diseases and uncovering hidden patterns to predict outcomes. This study aims to identify effective ML models for assessing the health conditions of children under five using the PDHS dataset, while also determining the most accurate model with the least error. A key focus is predicting the health status of unwanted children, a critical indicator of human development.

## Methods

In this section, we discuss the sources of data and inclusion criteria, response and explanatory variables of the study, principle component analysis (PCA) and multivariate analysis for child and treatment of child morbidity.

### Source of data and inclusion criteria

This study utilizes secondary data from the most recent PDHS conducted in 2017–18. The PDHS was designed to collect reliable data for monitoring the country’s population and health status. Its primary objectives include gathering information on fertility rates, FP practices, infant feeding methods, nutritional status, maternal and child health indicators, child mortality, women’s empowerment, domestic abuse, HIV/AIDS prevalence, population movement, disability statistics, and other health-related factors.

Our study specifically focuses on maternal and child health indicators collected during the postpartum period. The 2017–18 PDHS sampling strategy provides estimates at multiple geographical levels, including national, urban, and rural divisions, as well as provincial data for Punjab, Sindh, Khyber Pakhtunkhwa (KPK), and Balochistan. Additionally, it covers Azad Jammu and Kashmir (AJK), Gilgit Baltistan (GB), Islamabad Capital Territory (ICT), and the former Federally Administered Tribal Areas (FATA), totaling 13 secondary-level domains. However, it is important to note that national aggregate indicators do not include data from AJK and GB.

The study included interviews with a nationally representative sample of 12,364 women aged 15–49 who had been married at least once, residing in 12,815 selected households. Additionally, 3,145 men aged 15–49, also married at least once, were interviewed from one-third of these households.

The 2017–18 PDHS, part of the international DHS program, employed a two-stage stratified sampling methodology and achieved a response rate of 96%. The methodology section of the final report is available at https://dhsprogram.com/pubs/pdf/FR354/FR354.pdf, and the data-set can be accessed https://nips.org.pk/viewpublicdata. The sample is nationally representative, and data for this study was accessed on June 10, 2021.

Each woman in the study was asked about her childbirth experiences and preferences in the five years preceding the survey. Women who expressed a desire for more than nine children were excluded, as this number exceeds natural fertility levels and is often attributed to religious beliefs (e.g., “the number of children is entirely up to God”).

Additionally, we incorporated the PDHS variable “whether last pregnancy was wanted,” categorized as “wanted then,” “wanted later,” and “wanted no more,” in a sensitivity analysis related to unwanted children. This approach accounts for rationalization bias, recognizing that an initially unwanted child may become desired after birth. Furthermore, the authors did not have access to any personally identifiable information during or after data collection, ensuring participant confidentiality.

Co-variates in this study were selected based on existing literature and their established influence on child health, covering demographic, socioeconomic, spatial, and programmatic factors [6,40–42]. To ensure data quality and robustness, preprocessing included handling missing values and identifying outliers using the mean-variance standard deviation method. Categorical variables were encoded using one-hot or ordinal encoding, while binary variables were converted to 0 and 1. Feature scaling was applied where appropriate, and continuous variables such as maternal age were categorized based on standard classifications. The data-set was then split into training and testing sets to enable reliable model evaluation.

The study incorporates a range of health, demographic, socioeconomic, empowerment, spatial, and programmatic variables. Demographic factors include maternal age (grouped), year of birth, sex of the child, and birth order. Maternal education is classified as no education, primary, middle, or high school. Socioeconomic status is measured using the PDHS wealth index (poor, middle, rich). Women’s empowerment is assessed through decision-making on healthcare, major purchases, and family visits. Spatial variables include province (Punjab, KPK, ICT, Sindh, Baluchistan) and residence (urban or rural). Programmatic variables include antenatal care, postnatal checkups (PNC), FP worker visits in the last six months, and access to media (television, radio, newspapers), all recorded as binary indicators. Details of these variables are provided in the S1 Appendix.

### Response variable

The study’s outcome variables included PNC within two days of delivery, morbidity indicators such as diarrhea and fever, and treatments for conditions like cough, shortness of rapid breath (SRB), rapid breathing, and acute respiratory infection (ARI). Morbidity data was collected based on the presence of illness (yes or no) within the two weeks preceding the survey. Fever prevalence was determined by calculating the proportion of children under five who had a fever during this period. The same method was used to assess the prevalence of cough and SRB. Respiratory infection was measured by identifying the percentage of children exhibiting symptoms of short or rapid breathing. Beyond morbidity indicators, the study also examined newborn PNC within the first two days of birth, interventions for diarrhea, treatments for fever or cough, and vitamin A supplementation.

### Explanatory variable

This study aims to determine the prevalence of unwanted children based on various demographic and socio-economic factors and to explore ways to prevent it using ML techniques. Factors such as parity, maternal age, maternal education, and maternal income may significantly influence the likelihood of unwanted childbearing. These variables were carefully considered and controlled throughout the study to ensure a comprehensive analysis. The research focuses on identifying cases of excess childbearing, including instances where families have more boys, more girls, both genders in excess, or an overall higher-than-desired number of children (excess parity). Additionally, the study considers cases where no excess children were reported. A special emphasis is placed on the “desired status” category, which serves as the primary predictor of unwanted childbearing.

To assess these factors, women aged 45 and older who participated in the DHS were asked about their ideal number of children. This included questions regarding the preferred gender composition of their offspring, such as the number of boys, girls, or whether they had no specific preference for either gender. Based on these responses, four categories of unwanted children were identified: (a) an excess of male children, (b) an excess of female children, (c) an excess of both genders, and (d) an excess in overall parity (having more children than desired). These classifications help provide deeper insights into parental preferences and the factors contributing to unintended childbearing.

Additional factors influencing children’s health, including socioeconomic, geographic, and programmatic characteristics, were identified based on existing literature. Key demographic and health variables considered were maternal age at childbirth, child’s age, birth order (parity), and gender. Prior research has categorized women based on their age at motherhood; see [39] for details. Socioeconomic factors included maternal education (none, primary, medium, and higher), wealth index, employment status, women’s empowerment, and religion. These elements significantly impact maternal and child health by shaping access to healthcare, nutrition, and overall living conditions.

This study encompasses all provinces of Pakistan, including Punjab, Sindh, KPK, and Balochistan, along with ICT, the FATA, and both urban and rural regions. Additionally, PNCs were included as a key variable. Programmatic factors affecting child health in Pakistan were also considered, including women’s access to television, radio, newspapers, and magazines, as well as home visits by FP professionals within the past six months. The DHS classified PNC status for the most recent birth into three categories: “yes”, “no”, and “unknown”. Furthermore, education and wealth at the community level were used as indicators for assessing individual-level socio-economic conditions. The following table provides an overview of the explanatory variables, their definitions, and corresponding measurement units.

### Explanatory variables in measuring undesiredness

We assessed the undesirable children by using the standard methodology described in the literature. Te total number of live births (*LC*_*i*_) is the combination of boy (*B*_*i*_) and girl child (*G*_*i*_) of a mother provided in Eq (1). Based on a standard technique, the following are the indicators of unwanted children. Assume *C*_*i*_ represents the number of living children born to the same mother, including *i* who may or may not have *G*_*i*_ or *B*_*i*_.

$$C_{i}=B_{i}+G_{i}.$$ (1)

now, let consider the summary of the mother’s desired family preferences:

$$C_{m}=B_{m}+G_{m}+N_{m}$$ (2)

where *C*_*m*_ is the mother’s desired family size, (*B*_*m*_) is the number of boys, (*G*_*m*_) is the number of girls, and (*N*_*m*_) is the children of either gender preferred by mother.

The excess in parity is described by The excess in parity is indicated by *Ep*_*i*_ = 1, when the difference between the actual number of children *C*_*i*_ and the mother’s desired number of children *C*_*m*_ is greater than 0 (i.e., $Ep_{i}=C_{i}-C_{m}>0$).

Let the gender of the child *i* be represented as *S*, which can either be male or female. The number of children sharing the same sex as child *S*_*i*_ (*B*_*i*_ for boys or *G*_*i*_ for girls), along with the preference for children of that gender, *S*_*m*_, can then be determined. The resulting equation is as follows:

$$ES_{i}=1\;\text{if}\;ES_{i}=S_{i}-(S_{m}+N_{m})>0.$$ (3)

Children are considered in excess if either the total number of children or the number of children of the same gender exceeds the desired amount. For illustration, assume that a mother has six children and wishes to have four more—one girl, two boys, and one child of either gender to complete her desired family. She initially gave birth to four sons. The first three boys are not considered excessive. However, the fourth boy is excessive in terms of gender composition, but not in terms of total number of children (parity). Later, one of her sons passed away. She then had a girl, who was not considered excessive. However, the last child she gave birth to is considered excessive in both gender composition and total number of children.

### Principal component analysis

The PCA is a powerful statistical technique used to simplify a data table containing cases and variables by reducing it to its core features, called principal components. These components are linear combinations of the original variables that best explain the variance of all variables, see [43] and the references cited therein. From S2 Table (given in supplementary file), we can clearly see that we have 16 explanatory variables of different predictors (undesired predictor and socioeconomic and demographic predictor). From the group of aforementioned variable it is tedious to model and interpret the whole variable. So, in this study, we used PCA to extract key variables from a set of explanatory variables. Subsequently, these variables were incorporated into the multivariate model. Prior to conducting PCA, the dataset was organized to ensure an equal number of observations across all explanatory variables, given variations in observation counts.

### Multivariate probit model

The multivariate probit model (MPM) is used to regress a set of correlated binary variables on a combination of continuous and discrete predictors. While it has been applied in fields such as biology, economics, and psychophysiology, its use in medical research remains limited. We have demonstrated this model applicability and value in addressing medical problems. For more details, see [44] and references cited therein. The MPM is a specific case of a class of correlated prediction models. A correlated prediction model is especially useful when the goal is to predict or classify into diagnostic categories that consist of combinations of binary responses. [45] used data from the DHS of 22 Sub-Saharan African (SSA) countries to explore the relationships between household wealth and the incidence of fever, which serves as an indicator of malaria, among children in sub-Saharan Africa. This investigation employed both bivariate analysis and the MPM. The results show that both the occurrence of fever and its treatment are associated with poverty, as indicated by wealth, in SSA. In this study, we use MPM following the PCA to measure the undesiredness for the undesired child.

## Results

In this section, we discuss the summary statistics for variable given in S2 Table (S1 Appendix), results of undesired child based on particular socioeconomic characteristics, child morbidity, treatment of child morbidity, and demographic child morbidity in Pakistan.

### Statistical analysis

This section will discuss descriptive statistics for appropriate categorical types of variables. A simple uni-variate, non-graphical exploratory data analysis method for categorical variables is to build a Table 1 containing the fraction (or frequency) of data for each category.

**Table 1:** Descriptive statistics of explanatory variable.

Predictors	Mean	Variance	Standard Deviation	Skewness	Kurtosis
Excess in girls	0.2200	0.1712	0.4138	1.3588	2.8463
Excess in parity	0.1647	0.1377	0.3711	1.8081	4.2693
Excess in dual	0.2275	0.1759	0.4194	1.3000	0.6900
Excess in boys	0.1044	0.0936	0.3059	2.5872	7.6938
*edu*	0.9177	1.1971	1.0941	0.7124	2.0252
*exc*–*m*	0.1919	0.1552	0.3939	1.5652	3.4498
*empment*	0.1876	0.3361	0.5797	3.4407	14.729
*wealth*	1.8964	0.7913	0.8896	0.2037	1.2953
*bord*	1.4856	0.6321	0.3995	0.9428	2.8191
*resid*	1.5280	0.2494	0.4994	-0.1122	1.0126
*visit*–*hw*	0.5492	0.2478	0.4978	-0.1979	1.0392
*prov*	3.0365	4.3547	2.0868	1.1312	3.4033
*mage*	2.2301	0.3948	0.6283	0.1918	3.0808
*wsw*	0.1562	0.1319	0.3632	1.8940	4.5873
*age*–*ch*	1.9737	0.8192	0.9051	0.7740	2.9652
*sex*–*ch*	0.4626	0.2488	0.4988	0.1498	1.0224

Those summary statistics that were previously discussed contain data for frequency, mean, variance, standard deviation, kurtosis, and skewness. Table 1 displays an excerpt of the summary statistics about the demographic outcomes of unwanted children. If morbidity is the dependent variable, then variations in the degree of sickness among cohorts might fully or partially explain variations in mortality, determining these factors are crucial. Sample statistics describe the properties of a sample using a small number of factors. Typically, statisticians consider them as estimates of the corresponding population parameters from which the sample is drawn. These attributes can convey the data’s spread (variance, standard deviation, interquartile range, greatest and lowest value), central tendency (arithmetic mean, median, mode), or certain distributional properties (skewness, kurtosis). In above table, overall mean and variance of excess in girl is 22% and 17%, while excess in boys is 10% and 9%.

### Undesired child in Pakistan

Table 2 presents the number of unwanted offspring by a variety of the research population’s background variables. The majority of socioeconomic traits had a strong correlation with unwanted offspring. Adult women (aged 15–49) had the highest prevalence of unintended pregnancies. The excess of boys, girls, both boys and girls, and parity among adult mothers (aged 15–49) were 16.52%, 34.22%, 33.92%, and 28.61%, respectively. In the same way, the excess of boys, girls, boys, and girls together and parity among mothers over 49 were 25%, 45%, 40%, and 40%, respectively.

**Table 2:** Undesired children based on particular socioeconomic characteristics.

Background	Unit	Excess in boy	Excess in girl	Dual Excess	Excess in parity	Total number
*sex*–*ch*	Male	15.32	15.17	22.59	16.27	633
	Female	4.77	29.72	22.94	16.70	545
*mage*	Less then 20	0.00	9.26	6.48	0.00	108
	20-29	8.72	17.30	19.41	12.52	711
	30-39	16.52	34.22	33.92	28.61	339
	40-49	25.00	45.00	40.00	40.00	20
*resid*	Urban	11.69	22.12	26.98	18.88	556
	Rural	9.32	21.70	18.97	14.31	622
*prov*	Punjab	11.98	21.86	23.35	20.36	334
	Sindh	7.46	17.54	14.04	11.40	228
	KPK	12.33	26.43	34.80	18.94	227
	Baluchistan	11.27	26.06	19.01	15.49	142
	ICT	19.15	23.40	32.98	26.60	94
	FATA	3.88	25.24	20.39	9.71	103
*wealth*	Poor	9.94	23.45	17.45	14.07	533
	Middle	11.97	26.07	27.35	19.23	234
	Rich	10.22	17.52	27.01	18.00	411
*edu*	No education	10.39	22.73	20.45	14.45	616
	Primary	10.33	26.09	26.09	21.20	184
	Secondary	9.70	20.68	22.78	16.88	237
	Higher	12.06	14.89	28.37	18.44	141
*exc*–*m*	No	10.50	21.43	21.22	15.34	952
	Yes	10.18	23.89	29.20	21.24	226
*wsw*	No	10.06	21.43	21.73	15.09	994
	Yes	12.50	24.46	28.26	23.91	184

Unwanted children are more common among mothers in metropolitan settings. The idea that impoverished and illiterate women were more likely than others to have unwanted children was both intriguing and believable. Likewise, women who have access to any form of social media are more likely than others to become pregnant with an unwanted child. Compared to women who do not work, working women bear more unwanted children.

### Child morbidity

Table 3 presents the child morbidity and treatment of child morbidity of undesired children in Pakistan. We discovered that in the two weeks prior to the survey, 11.21%, 19.53%, 24.18%, and 17.78% of the undesirable predictors—excess in boys, excess in girls, dual excess, and excess in parity, respectively—had diarrhea. 11.15% excess in boys, 19.89% excess in girls, 24.23% dual excess, and 16.46% of the children had fever in the last two weeks. Similarly, 10.88% of the boys, 19.40% of the girls, 23.61% of the dual excess, and 16.01% of the children had coughs in the last two weeks. Morever, from Table 3, 10.27% excess in boys, 18.55% excess in girls, 22.64% of dual excess, and 15.21% of the children had SRB problems. 10.44% excess in boys, 21.90% excess in girls, 22.75% dual excess, and 16.47% of the children had ARI problems.

**Table 3:** The ideal status of children in Pakistan and the prevalence (%) of illness among newborns in the previous five years.

Outcome	Excess in boy		Excess in girl		Dual excess		Excess in parity		
	No	Yes	No	Yes	No	Yes	No	Yes	Total
**Child morbidity**									
Diarrhea	88.79	11.21	80.47	19.53	75.82	24.18	82.22	17.78	6253
Fever	88.85	11.15	80.11	19.89	75.77	24.23	83.54	16.46	6153
Cough	89.12	10.88	80.60	19.40	76.39	23.61	83.99	16.01	6422
SRB	89.73	10.27	81.45	18.55	77.36	22.64	84.79	15.21	3525
ARI	89.56	10.44	78.10	21.90	77.25	22.75	83.53	16.47	1178
**Treatment of child**									
**morbidity**									
PNC	90.03	9.97	82.17	17.83	78.20	21.80	85.41	14.59	3702
Vitamin A	89.73	10.27	81.45	18.55	77.36	22.64	84.79	15.21	3525
ToD	88.85	11.15	80.43	19.57	75.77	24.23	82.19	17.81	6153
ToF/C	88.47	11.31	76.67	20.58	77.24	24.06	83.21	16.77	2515

Note: ToD and ToF/C are abbreviated as treatment of diarrhea and treatment of fever/cough, respectively.

**Fig 1:**
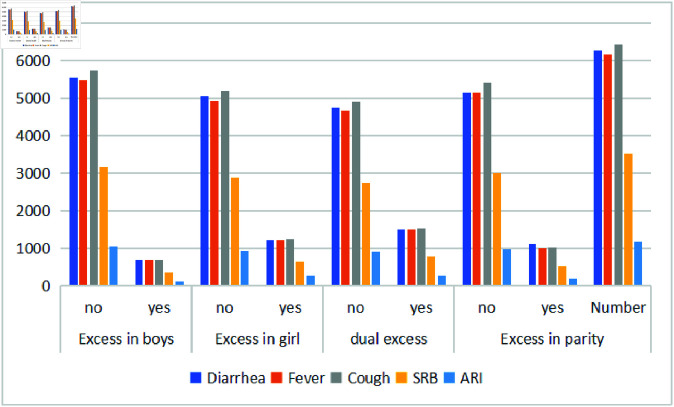
Causes of morbidity among unwanted children under the age of five.

Fig 1, presents the distribution of five child health conditions—diarrhea, fever, cough, SRB, and ARI—in Pakistan. Approximately 22%, 22%, 21%, 20%, and 22% of children experienced these conditions, respectively, in the past two weeks, with ARI showing the lowest prevalence. The figure also illustrates variations across categories of excess (boys, girls, dual, and parity). Diarrhea, fever, and cough are the most common across all categories, with higher prevalence observed in cases without demographic excess. SRB shows greater variation, while ARI consistently has the lowest counts. These patterns suggest possible associations between demographic excess and child morbidity, warranting further investigation.

### Postnatal care and treatment for childhood morbidity

**Fig 2:**
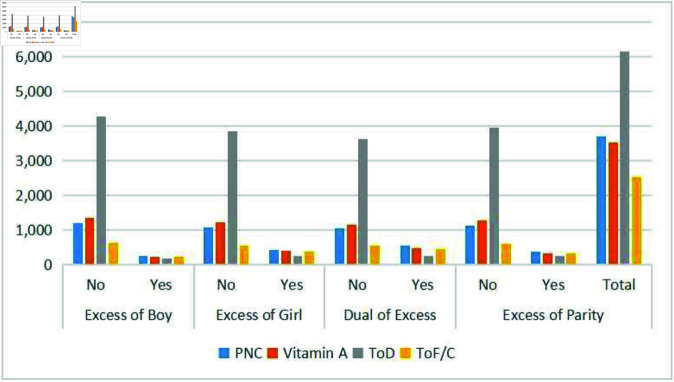
Frequency of morbidity treatments among unwanted children under the age of five.

Fig 2 shows medical treatment for common diseases and postnatal checks for neonatal within two weeks after birth. Within two days of delivery, 28% of children got postnatal care; this percentage was lower for wanted children. In contrast, 46% of the kids had diarrhea treatment within the previous six months. Regarding immunization, 26% of respondents got vitamin A in the six months before the survey; the percentage was lower for the children who were not selected.

### Demographic child morbidity

**Fig 3:**
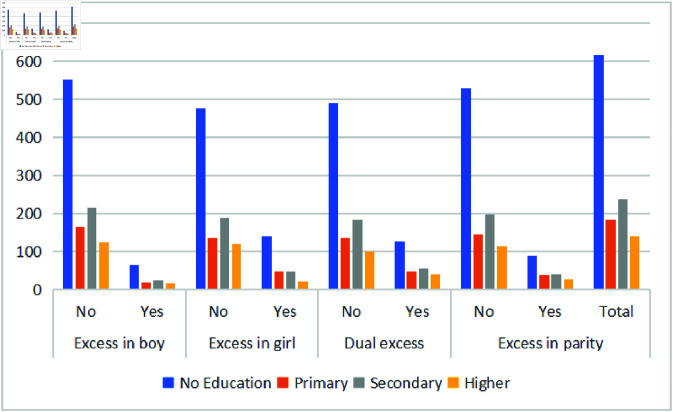
Morbidity cases attributable to unwanted children under the age of five by mother’s educational attainment.

**Fig 4:**
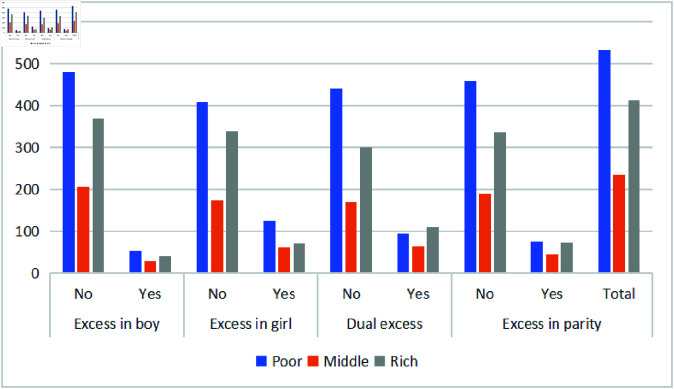
Frequency of under-five deaths among unwanted children by household wealth index.

Child morbidity, mortality, and malnutrition are closely associated with the mother’s socioeconomic status. Figs 3 and 4 illustrate under-five mortality rates among unwanted children, stratified by maternal education and household wealth status. Maternal education plays a critical role, with higher education levels linked to lower under-five mortality.

Mothers with no education account for 52% of child morbidity, while those with primary, secondary, and higher education contribute 16%, 20%, and 12%, respectively. Regardless of education level, the impact of unwanted children on mortality remains higher among boys, girls, and higher parity. Similarly, unwanted children show higher mortality across all wealth levels. Fig 4 shows that 45% and 35% of child morbidity in all undesired predictors occur in poor and rich households, respectively, while morbidity is lower among middle-income families.

**Fig 5:**
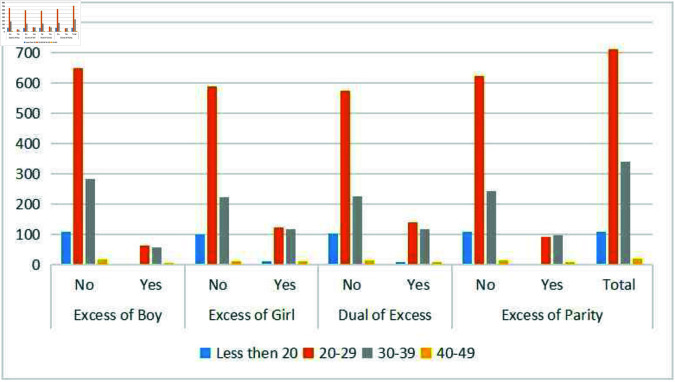
Frequency of under-five deaths among unwanted children by mother’s age.

Fig 5 illustrates child morbidity by maternal age. Mothers under 20 account for 9% of cases, those aged 20–29 for 60%, and those aged 30–39 for 29%. The highest morbidity is observed in the 20–29 age group, likely reflecting higher birth rates and exposure to risk factors. Additionally, excess in boys and parity is more prominent than excess in girls or dual excess, suggesting a potential link between male children, higher birth order, and increased morbidity. These patterns highlight the importance of addressing gender and parity-related health disparities.

**Fig 6:**
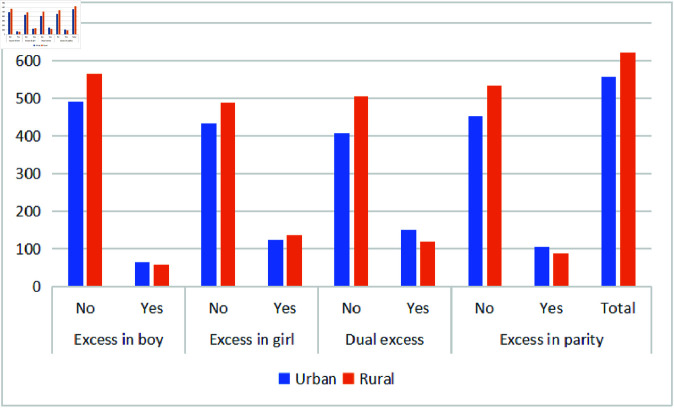
Prevalence of unwanted children under age five across residential.

**Fig 7:**
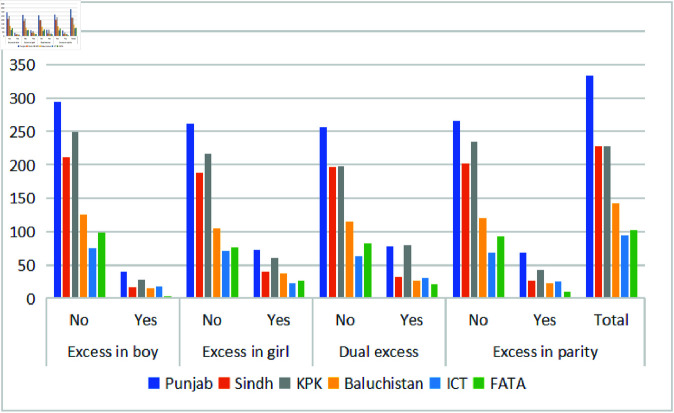
Provincial distribution of morbidity in unwanted children under five.

Figs 6 and 7 present under-five child morbidity rates attributed to unwanted children by maternal residence and province. The analysis covers districts in KPK, including the newly merged areas formerly known as FATA, as well as Punjab, Sindh, and Baluchistan. Maternal place of residence is a key factor influencing child health outcomes. Urban areas exhibit lower under-five mortality rates compared to rural regions, with 47% of child morbidity occurring in urban areas and 53% in rural areas. Additionally, excess in boys and dual excess are more prevalent in urban settings, indicating possible disparities linked to gender and residence.

The child morbidity is higher in Punjab with respect to other provinces of Pakistan, including FATA and ICT. The reason for the high morbidity is because of the population residing in Punjab. 52.90% of Pakistan’s population lives in this province, and the remaining population lives in other provinces. It is interesting to note that KPK has a higher boy rate in comparison to its population. Similarly, the excess in parity is higher in KPK when compared to other provinces except Punjab. Overall, 13–30% of the child morbidity in all undesired predictors is in Punjab. Similarly, Sindh and KPK share 20% of the child morbidity.

Regardless of educational achievement, the disproportional impact of undesired pregnancies on under-five mortality was more significant for both boys and girls, as well as across different parity levels. The household wealth index also showed comparable outcomes, indicating a higher death rate for unwanted children compared to desired children. From Table 4, it can be seen that 45% and 35% of the child morbidity in all undesired predictors are due to poor and rich families, respectively. It can be seen that in middle-class families, child morbidity is lower.

**Table 4:** The ideal status of children in Pakistan and the prevalence (%) of illness among newborns in the previous five years.

Child		Excess in boy		Excess in girl		Dual excess		Excess in parity		
Morbidity	Attribute	No	Yes	No	Yes	No	Yes	No	Yes	Total
*edu*	No Education	0.90	0.10	0.77	0.23	0.80	0.20	0.86	0.14	616
	Primary	0.90	0.10	0.74	0.26	0.74	0.26	0.79	0.21	184
	Secondary	0.90	0.10	0.79	0.21	0.77	0.23	0.83	0.17	237
	Higher	0.88	0.12	0.85	0.15	0.72	0.28	0.82	0.18	141
*wealth*	Poor	90.06	9.95	76.55	23.45	82.55	17.45	85.93	14.07	533
	Middle	88.03	11.97	73.93	26.07	72.65	27.35	80.77	19.23	234
	Rich	89.78	10.22	82.48	17.52	72.99	27.01	82.00	18.00	411
*mage*	Less than 20	100.00	0.00	90.74	9.26	93.52	6.48	100.00	0.00	108
	20-29	91.28	8.72	82.70	17.30	80.59	19.41	87.48	12.52	711
	30-39	83.48	16.52	65.78	34.22	66.08	33.92	71.39	28.61	339
	40-49	75.00	25.00	55.00	45.00	60.00	40.00	60.00	40.00	20
*resid*	Urban	88.31	11.69	77.88	22.12	73.02	26.98	81.12	18.88	556
	Rural	90.68	9.32	78.30	21.70	81.03	18.97	85.69	14.31	622
*prov*	Punjab	88.02	11.98	78.14	21.86	76.65	23.35	79.64	20.36	334
	Sindh	92.54	7.46	82.46	17.54	85.96	14.04	88.60	11.40	228
	KPK	109.69	12.33	95.59	26.43	87.22	34.80	103.08	18.94	227
	Baluchistan	88.73	11.27	73.94	26.06	80.99	19.01	84.51	15.49	142
	ICT	80.85	19.15	76.60	23.40	67.02	32.98	73.40	26.60	94
	FATA	96.12	3.88	74.76	25.24	79.61	20.39	90.29	9.71	103

## Uni-variate analysis for child and treatment of child morbidity

The ML techniques are popular in detecting the disease, extracting the pattern, and have the potential to improve efficiency; see [46] for details. Support vector machines, RF, NN, K-Nearest Neighbor, and decision trees are some popular ML algorithms that are extensively used in disease prediction. In this study, we used NN and RF for measuring child morbidity and their treatment. The goal of the aforesaid algorithms is to predict the health status of under-five children in Pakistan and disease. In addition, we analyzed the algorithms systematically using accuracy, sensitivity, and specificity.

### Child morbidity using RF and NN

In the following section, we discuss the RF and NN models for child morbidity in details given in subsection. Moreover, we also discuss the important variables for child morbidity and performance of RF and NN on child morbidity are also discussed in the subsection.

#### Random forest.

The RF is one of the most effective ML models for predictive analytics, making it an industrial workhorse for ML. The RF model is a type of additive model that makes predictions by combining decisions from a sequence of base models. More formally we can write this class of models as:

$$g(x)=f_{0}(x)+f_{1}(x)+f_{2}(x)+\cdots$$ (4)

where the final model *g*(*x*) is the sum of simple base models *f*_*i*_. Here, each base classifier is a simple decision tree. This broad technique of using multiple models to obtain better predictive performance is called model ensembling. In RF, all the base models are constructed independently using a different sub-sample of the data.

#### Artificial neural network.

Artificial NN (ANN), also known as a NN, has made significant progress. The ANN can handle several problems in a scientific discipline. In many fields, the most precise and popular forecasting models, including engineering, finance, social, business, economic, and foreign exchange rates, are ANNs as a soft computing method see [47] and the references cited therein for more details. Its distinctive qualities draw scholars and industry practitioners interested in forecasting time series data. In this technique, three layers are used, one is the input layer, which is of data entry; the other one is the processing of data which takes place in the middle layer; and the third layer is the output layer which provides results, see [48] for more detail. The mathematical form of ANN is given by:

$$M_{j}=\left(\sum_{k=1}^{j-1}X_{k}^{j-1}W_{k,j}-bk\right)$$ (5)

where $X_{k}^{j-1}$ denotes the input from k*th* node in the j*th* layer, *W*_*k*,*j*_ is the weight of the link between node *k* and all the nodes in the previous layers, and *bk* is the bias to the node and *N*_*j*−1_ is the number of nodes in the layer *j*–1. This sum is passed along to an activation function, to produce the output of the node calculated as follows: $Y_{i}=f(M_{i})$. The sigmoidal function is the most commonly used activation function, defined as:

$$f(M_{i})=\frac{1}{1+e^{-M_{i}}}$$ (6)

#### Important variables of child morbidity.

The primary objective of this study is to identify the most influential variables contributing to undesired child morbidity and mortality. The RF models typically assess variable importance using two key metrics: mean decrease in accuracy and mean decrease in Gini. The mean decrease in accuracy measures the reduction in model performance when a specific variable is excluded, indicating its significance in accurate classification. The mean decrease in Gini evaluates how much each variable contributes to the homogeneity of nodes and leaves within the RF model, offering insights into the relative importance of predictors. For a detailed explanation of the mean decrease in Gini and its role in variable selection, see [49] and related references.

**Fig 8:**
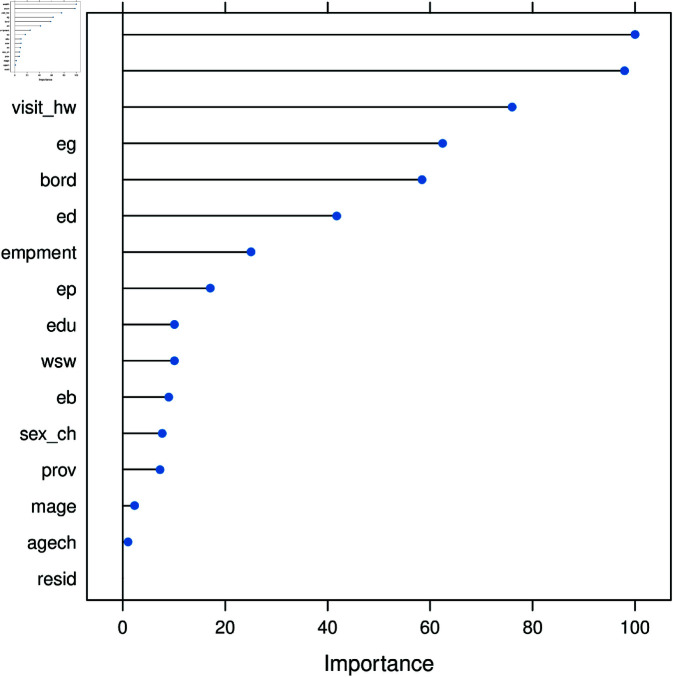
Important variables for ARI using a NN model.

**Fig 9:**
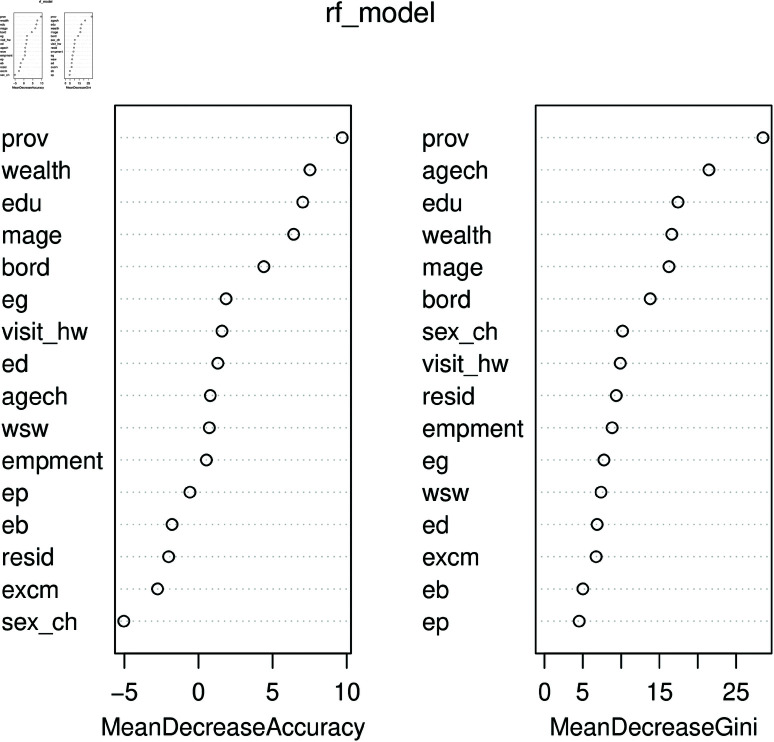
Important variables for ARI using a RF model.

Fig 8 illustrates that wealth and media access are key variables influencing ARI. Meanwhile, Fig 9 shows that under the mean decrease in accuracy method, province and wealth are important predictors, whereas under the mean decrease in Gini method, province and child’s age emerge as significant factors for ARI. Additionally, Fig 8 results were obtained using a NN model, while Fig 9 was derived from the RF model. The results for both methods using the RF model for identifying important variables are also presented in Table 5.

**Table 5:** Important variables (%) in child morbidity using RF model.

	Mean Decrease Accuracy				Mean Decrease Gini			
Outcome	Var-1	Imp	Var-2	Imp	Var-1	Imp	Var-2	Imp
Cough	*wealth*	17.7	*edu*	17.9	*prov*	07.1	age ch	10.7
Fever	*wealth*	43.0	*edu*	36.0	*prov*	35.0	age ch	48.3
Diarrhea	*wealth*	16.5	*edu*	30.0	*prov*	13.0	age ch	4.20
ARI	*wealth*	30.5	*edu*	23.0	*resid*	29.0	age ch	4.20
SRB	*wealth*	21.0	*resid*	22.0	*edu*	78.0	*prov*	16.0

Note: Var and Imp are abbreviated as variable and important, respectively.

The incidence of cough symptoms in undesired children is higher among middle-income (17.7%) and uneducated (17.9%) mothers. The mean decrease in Gini index highlights province and child age as key predictors. As shown in Fig 3, female education (36%) and household wealth (43%) are significant determinants of ARI in undesired children. Furthermore, women from Baluchistan and KPK report a 35% higher rate of ARI among girls.

#### Performance of RF and NN on child morbidity.

From Table 6, the RF is 96.27% accurate for fever, has a 99% sensitivity rate, and a 92% specificity rate. On the other hand, the NN model accurately predicted 64.5% of children who had a fever during the previous two weeks. Given that NN has an accuracy of 77.65% with 8% sensitivity and a specification of 95%, and RF has an accuracy of 79.2%, the prevalence of SRB in children under 5 is greater.

**Table 6:** Performance of RF and NN algorithms on child morbidity.

Model	Measure	Fever	Cough	Diarrhea	ARI	SRB
RF	accuracy	96.27	62.00	77.00	75.00	79.20
	sensitivity	99.40	90.24	01.00	08.54	99.11
	specificity	92.13	09.09	00.00	96.20	00.70
NN	accuracy	64.50	64.40	77.50	70.45	77.65
	sensitivity	28.42	30.60	01.99	00.00	08.20
	specificity	77.90	67.00	98.60	13.63	95.93

Nonetheless, a study by [39] revealed that there was an increase in ARI among boys; the RF model indicated an accuracy of 75%, while the NN model indicated an accuracy of 70.45%. In contrast to [50], the undesired cough within the last two weeks demonstrated higher accuracy. However, NN performed better with 64.4% sensitivity and 68% specification for children with coughs, while RF performed worse with 62% accuracy and 90% sensitivity. When predicting the ARI among the unwanted kids, RF’s algorithm performs the best, with an accuracy of 77%, 1% sensitivity, and 0% specification. When it comes to diarrhea, NN reports that the prevalence of diarrhea in the past two weeks is higher—77.5%—among the undesirable children, whereas RF fared worst.

### Treatment of child morbidity using RF and NN

In the following section, we discuss the important variables for treatment of child morbidity and performance of RF and NN on treatment of child morbidity are also discussed in the subsection.

#### Important variables of treatment of child morbidity.

**Fig 10:**
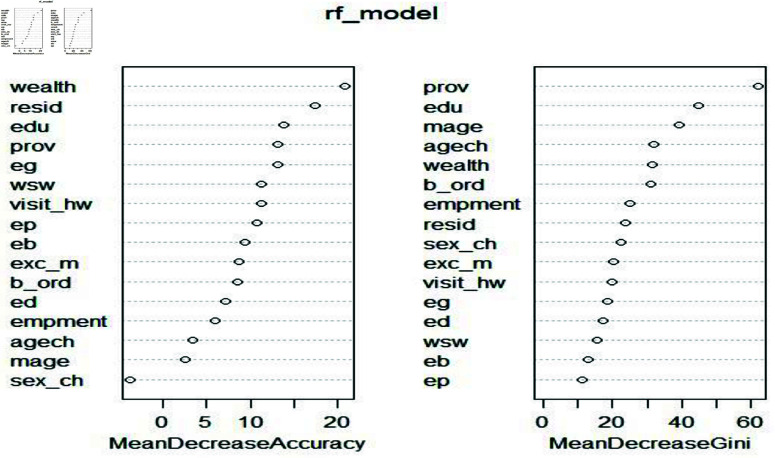
Important variables for PNC using a RF model.

The Table 7 presents the importance of variables in predicting child morbidity outcomes using RF. The “Mean Decrease Gini” and “Mean Decrease Accuracy” values indicate the relative importance of each variable for different outcomes, with “prov” and “wealth” being consistently important across several outcomes. Additionally, variables such as “edu” and “age ch” also show significant importance for certain outcomes like vitamin A and ToD. Moreover, Fig 10 shows that 5.1% of boys receive less treatment for diarrhea, with 23% of cases linked to undesired children and 40.92% associated with uneducated mothers from Baluchistan. Child age and household wealth are key factors influencing diarrhea treatment. Additionally, girls from poor and middle-income families in the FATA and KPK regions receive the least treatment for fever or cough, with child age being a significant contributing factor.

**Table 7:** Important variables in treatment of child morbidity using RF.

	Mean Decrease Gini				Mean Decrease Accuracy			
Outcome	Var-1	Imp	Var-2	Imp	Var-1	Imp	Var-2	Imp
PBC	*prov*	68.45	*wealth*	67.78	*wealth*	48.77	*edu*	42.42
Vitamin A	*edu*	56.82	*mage*	53.56	*prov*	40.53	*wealth*	13.45
ToD	*prov*	40.92	age ch	33.35	*wealth*	30.90	*edu*	23.00
ToF/C	*prov*	86.69	age ch	59.69	*wealth*	19.27	*resid*	14.07

The RF enables us to assess feature importance, which reflects the extent to which the Gini index for a feature contributes to the model. The undesired girls born to poor mothers (48.77%) are less likely to receive any type of postnatal care. Women’s education (42.4%) and province (68.45%) are two significant factors associated with receiving the lowest postnatal care among undesired children.

#### Performance of RF and NN on treatment of child morbidity.

Efforts were made to assess the impact of undesired children on the treatment of child morbidity. Results in Table 8 show that the postnatal check using testing data from the NN shows an accuracy of 52.9% with 63.4% sensitivity and a specification of 35%. The NN performance is worse in the case of postnatal baby check as compared to other variables.

**Table 8:** Performance of RF and NN algorithms on treatment of child morbidity.

Model	Measure	ToD	PBC	Vitamin A	ToF/C
RF	accuracy	72.25	63.36	72.47	63.92
	sensitivity	47.62	17.77	17.00	49.10
	specificity	85.47	87.20	92.50	74.45
NN	accuracy	69.70	52.90	67.90	62.80
	sensitivity	82.69	63.40	92.20	65.06
	specificity	45.50	35.80	57.00	59.70

For cases where diarrhea has been treated in the past two weeks, the RF model achieves an accuracy of 72.25%, with a sensitivity of 47.62% and a specificity of 85.47%. In contrast, the NN model has an accuracy of 69.7%, with a sensitivity of 82.69% and a specificity of 45.5%. With a sensitivity of 49% and a specification of 74%, the RF accuracy for treating cough or fever within the past two weeks is 63.9%. With a 65% sensitivity and a 57% specification, the NN accuracy is 62.8%.In the context of receiving vitamin A within 6 months, the RF model outperforms the other classifier.

## Multi-variate analysis for child morbidity

In this section, we specific discus the correlation matrix, PCA and multi-variate analysis only for child morbidity. However, the aforementioned analysis are precluded for the treatment of child morbidity.

### Correlation matrix of child morbidity for explanatory variable

**Fig 11:**
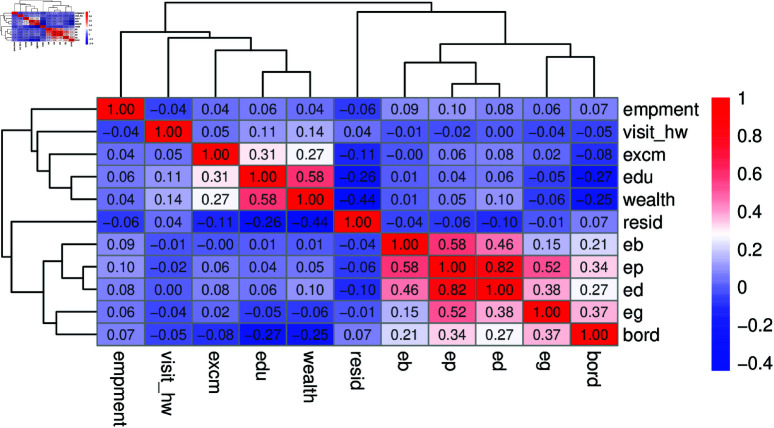
Correlation matrix of explanatory variables.

A multiple binary correlation matrix displays correlation coefficients between binary variables, helping to understand their pairwise relationships. For binary data, the tetrachoric correlation is often used, as it estimates the association between underlying continuous variables assumed to follow a bi-variate normal distribution. As shown in Fig 11, *eg*, *ed*, *ep*, and *eb* are positively correlated, as are *edu* and *wealth*.

### Principal component analysis

**Fig 12:**
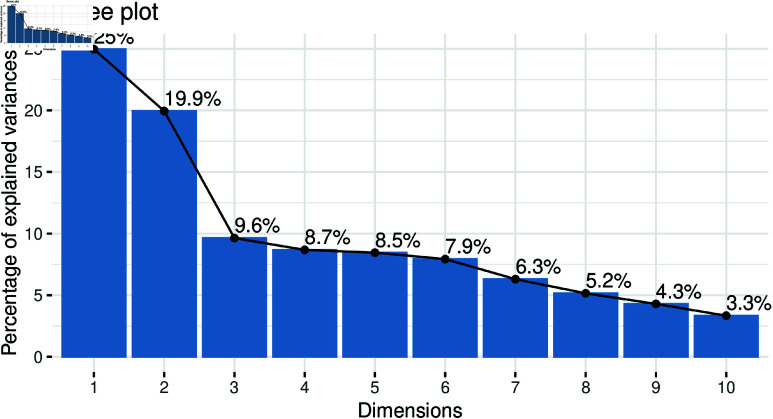
Variance explained by each principal component.

S2 Table (supplementary file) lists 16 explanatory variables covering undesired, socioeconomic, and demographic predictors. Modeling all variables directly is complex; thus, PCA was applied to reduce dimensionality and identify key features. Prior to PCA, assumptions were assessed: the correlation matrix (Fig 11) confirmed sufficient inter-variable relationships, and both Bartlett’s Test of Sphericity and the Kaiser-Meyer-Olkin (KMO) measure indicated data suitability (KMO = 0.675). As the variables were binary, standardization was not applied before these tests. PCA simplifies data by transforming correlated variables into principal components—linear combinations that capture maximum variance—enabling more interpretable modeling. Fig 12 presents the PCA results, including the scree plot and other visualizations.

**Fig 13:**
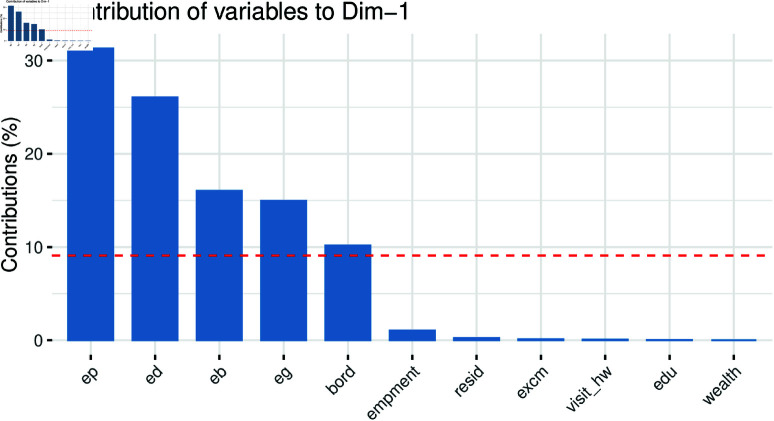
Individual variable contribution to principal component.

In a scree plot, each principal component is shown as the percentage of variance it explains, helping to determine the number of components to retain. The “elbow” point—where the explained variance begins to level off—indicates the optimal number of components. As shown in Fig 13, the optimal components identified are ep,ed,eg,eb, and *bord*. To implement a MPM, we first perform PCA on the explanatory variables, which consist of 16 features. After conducting PCA, we select the most important components based on their explanatory power. These selected components are then utilized as the explanatory variables for the MPM. It is important to note that, in MPM, all variables must have an equal number of observations.

### Multivariate probit model

The MPM is an extension of the standard probit model designed to handle multiple correlated binary outcomes simultaneously. It is particularly useful in situations where the binary outcomes are not independent, allowing for the capture of correlations between them. The model is used when there are several binary dependent variables, and the interest lies in understanding their joint behavior. Each binary outcome is associated with a latent continuous variable, which follows a multivariate normal distribution.

Let $y_{ij}^{*}$ is a latent variable for the *j*th binary outcome of the *i*th observation, defined as:

$$y_{ij}^{*}=X_{i}\beta_{j}+\epsilon_{ij},$$ (7)

where *X*_*i*_ is a vector of explanatory variables, $\beta_{j}$ is a vector of coefficients, and $\epsilon_{ij}$ follows a multivariate normal distribution with mean zero and covariance matrix Σ. The binary outcome *y*_*ij*_ is derived from the latent variable: 1 if $y_{ij}^{*}>0$, and 0 for otherwise. The MPM provides a robust framework for analyzing correlated binary outcomes, offering insights that are not available from separate uni-variate probit models. The MPM is useful in various applications, such as modeling multiple binary health outcomes and analyzing survey data with multiple binary responses.

**Fig 14:**
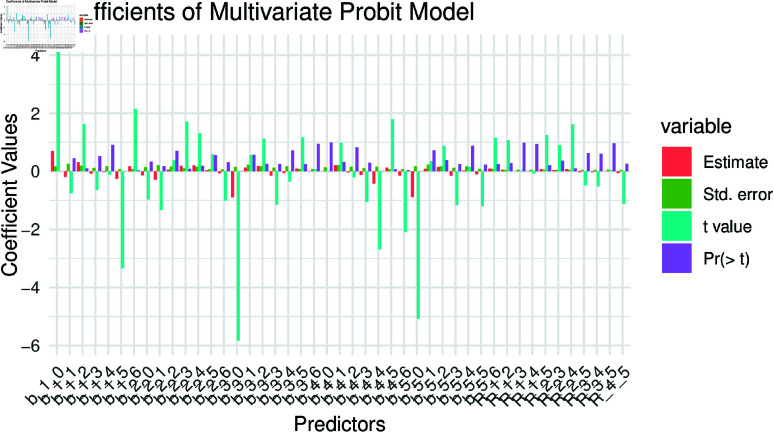
Coefficients of MVP model on child morbidity.

It should be noted that from Fig 14 and Table 9, *b*–1–0 corresponding to intercept of ARI holding the explanatory variable constant. Similarly, *b*–1–1, *b*–1–2, *b*–1–3, *b*–1–4, *b*–1–5, and *b*–1–6, are the beta coefficients between ARI and *ep*, ARI and *ed*, ARI and *eg*, ARI and *eb*, ARI and *bord*, and ARI and *mage*, respectively. Similarly, *b*–2–0, *b*–2–1, *b*–2–2, *b*–2–3, *b*–2–4, *b*–2–5, and *b*–2–6, corresponding to intercept of cough and the beta coefficients between cough and *ep*, cough and *ed*, cough and *eg*, cough and *eb*, cough and *bord*, and cough and *mage*, respectively. On the similar lines, one can interpret the other intercept and coefficients given in Fig 14 and Table 9, respectively.

**Table 9:** Coefficients of MVP model on child morbidity.

Coefficents	Meaning	Estimate	Std.Error	t values	Pr(>t)
b_1_0	Intercept ARI	0.7034	0.1674	4.2020	0.0000
b_1_1	Coef. between ARI and *ep*	-0.1954	0.2587	-0.7550	0.4501
b_1_2	Coef. between ARI and *ed*	0.3191	0.1969	1.6210	0.1051
b_1_3	Coef. between ARI and *eg*	-0.0785	0.1224	-0.6420	0.5211
b_1_4	Coef. between ARI and *eb*	-0.0196	0.1790	-0.1090	0.9130
b_1_5	Coef. between ARI and *bord*	-0.2586	0.0775	-3.3350	0.0009
b_1_6	Coef. between ARI and *mage*	0.1720	0.0799	2.1510	0.0314
b_2_0	Intercept Cough	-0.1375	0.1428	-0.9630	0.3357
b_2_1	Coef. between Cough and *ep*	-0.2869	0.2155	-1.3320	0.1829
b_2_2	Coef. between Cough and *ed*	0.0603	0.1610	0.3740	0.7082
b_2_3	Coef. between Cough and *eg*	0.1903	0.1113	1.7100	0.0873
b_2_4	Coef. between Cough and *eb*	0.2045	0.1559	1.3120	0.1896
b_2_5	Coef. between Cough and *bord*	0.0423	0.0728	0.5810	0.5611
b_2_6	Coef. between Cough and *mage*	-0.0694	0.0689	-1.0060	0.3143
b_3_0	Intercept Diarrhea	-0.8941	0.1533	-5.8320	0.0000
b_3_1	Coef. between Diarrhea and *ep*	0.1294	0.2295	0.5640	0.5729
b_3_2	Coef. between Diarrhea and *ed*	0.1870	0.1661	1.1260	0.2601
b_3_3	Coef. between Diarrhea and *eg*	-0.1462	0.1273	-1.1480	0.2509
b_3_4	Coef. between Diarrhea and *eb*	-0.0605	0.1711	-0.3540	0.7236
b_3_5	Coef. between Diarrhea and *bord*	0.0934	0.0805	1.1610	0.2458
b_3_6	Coef. between Diarrhea and *mage*	0.0049	0.0742	0.0660	0.9472
b_4_0	Intercept Fever	-0.0008	0.1421	-0.0050	0.9958
b_4_1	Coef. between Fever and *ep*	0.2104	0.2150	0.9790	0.3277
b_4_2	Coef. between Fever and *ed*	-0.0327	0.1571	-0.2080	0.8351
b_4_3	Coef. between Fever and *eg*	-0.1209	0.1153	-1.0490	0.2944
b_4_4	Coef. between Fever and *eb*	-0.4220	0.1569	-2.6890	0.0072
b_4_5	Coef. between Fever and *bord*	0.1303	0.0726	1.7940	0.0728
b_4_6	Coef. between Fever and *mage*	-0.1457	0.0696	-2.0920	0.0364
b_5_0	Intercept SRB	-0.8822	0.1739	-5.0730	0.0000
b_5_1	Coef. between SRB and *ep*	0.0812	0.2301	0.3530	0.7241
b_5_2	Coef. between SRB and *ed*	0.1502	0.1718	0.8750	0.3818
b_5_3	Coef. between SRB and *eg*	-0.1480	0.1269	-1.1660	0.2435
b_5_4	Coef. between SRB and *eb*	0.0256	0.1703	0.1510	0.8803
b_5_5	Coef. between SRB and *bord*	-0.0981	0.0818	-1.1990	0.2303
b_5_6	Coef. between SRB and *mage*	0.0954	0.0828	1.1530	0.2490
R_1_2	Corr. between ARI and Cough	0.0562	0.0525	1.0710	0.2840
R_1_3	Corr. between ARI and Diarrhea	0.0010	0.0577	0.0170	0.9863
R_1_4	Corr. between ARI and Fever	-0.0039	0.0527	-0.0750	0.9404
R_1_5	Corr. between ARI and SRB	0.0747	0.0595	1.2550	0.2096
R_2_3	Corr. between Cough and Diarrhea	0.0462	0.0515	0.8980	0.3690
R_2_4	Corr. between Cough and Fever	0.0760	0.0469	1.6200	0.1053
R_2_5	Corr. between Cough and SRB	-0.0254	0.0527	-0.4820	0.6295
R_3_4	Corr. between Diarrhea and Fever	-0.0269	0.0521	-0.5150	0.6062
R_3_5	Corr. between Diarrhea and SRB	0.0026	0.0584	0.0450	0.9640
R_4_5	Corr. between Fever and SRB	-0.0598	0.0535	-1.1170	0.2638

Note: Coef. and Corr. are abbreviated as Coefficient and Correlation, respectively.

From Table 9, the coefficient between ARI and *bord*, the explanatory variable *bord* has a negative and statistically significant effect on the outcome variable ARI (i.e., p value is less than 5%). Similarly, the coefficient between ARI and *mage*, the explanatory variable *mage* has a positive and statistically significant impact on the ARI. The coefficient between fever and *eb* and fever and *mage*, the independent variables *eb* and *mage* has negative and statistically significant effects on the Fever. Apart from these regression coefficients, all the other coefficients has insignificantly effect on the there respective outcome variables.

## Discussion

Globally, child mortality and morbidity are major concerns, especially in developing countries like Pakistan. It is also the biggest issue in nations with low and medium incomes. Two significant findings of this study are the treatment of child illness and morbidity across Pakistani study districts. The research also looked at how socio-demographics, housing, health, and environmental factors affected child mortality and morbidity in every district in Pakistan. Another goal of the study is to recommend to the population welfare department and relevant ministries how to stop the increasing trend of unwanted pregnancies by educating the public using specific print, electronic, and social media platforms. In this manner, taking into account the demographic and socioeconomic determinants, the rates of child morbidity and mortality might also be decreased.

Furthermore, between 2016 and 2030, the United Nations recognized the need to end preventable newborn and infant mortality, according to the world health organization (WHO). The third sustainable development goal (SDG) is to ensure and promote healthy lifestyles and well-being. According to the SDGs, by 2030, all nations should lower under-five moralities and neonatal mortality to 12 or fewer and 25 or fewer per 1000 live births, respectively. 118 nations’ under-five mortality rate was already below the SDG objective. However, for the remaining nations—mostly those in sub-Saharan Africa and central and southern Asia—to meet the goal, promises and development must quicken, according to [51]. Thus, lowering the rate of unintended pregnancies and childbirth will aid in achieving the objective.

We utilized secondary data from the PDHS 2017-18, which employed a two-stage stratified sampling methodology with a 96% response rate, ensuring national representativeness. To control for potential confounding variables, we excluded women desiring more than nine children, as this was considered beyond natural fertility. Additionally, we controlled for socio-economic factors such as mother’s education, income, and region to isolate the impact of unwanted pregnancies on child health outcomes. For replication, we employed two ML techniques, RF and NNs, to validate our findings, ensuring robustness. We also conducted sensitivity analyses by testing different thresholds for defining unwanted pregnancies and assessing child morbidity and mortality, which confirmed the consistency of our results. Finally, we used PCA to reduce dimensionality and then performed MPM. Additionally, the MPM allowed us to analyze the joint probabilities of various health outcomes (such as morbidity and mortality) associated with unwanted children.

In this study, multivariate analysis and PCA were applied to a dataset consisting of 12,364 observations. Given the substantial sample size and data completeness, the statistical methods used did not require additional assumptions to be made or explicitly tested. The large sample size provides a robust foundation for the analyses, as it inherently minimizes the effects of variability, outliers, or other potential anomalies in the data. Furthermore, the absence of missing data further strengthens the validity of the applied methods, ensuring that the results are not influenced by incomplete information. As a result, the analyses were performed directly on the full data-set without the need for imputation or assumption testing, making the results particularly robust. This approach highlights the advantages of large sample sizes in improving the precision and accuracy of statistical estimates. These considerations underscore the strength of our analysis, which benefits from the combination of a large, complete data-set and the appropriate use of multivariate techniques.

The analysis of unwanted children in Pakistan highlights several key findings regarding child morbidity and treatment patterns. The data shows that unwanted children, especially those with excess boys, girls, or higher parity, have a higher likelihood of experiencing common childhood illnesses, such as diarrhea, fever, cough, and ARI. These health issues are more prevalent among children born to mothers from poorer socioeconomic backgrounds, with lower education levels, and in rural areas. Interestingly, urban mothers have a higher rate of unwanted pregnancies, but children in rural areas experience greater overall morbidity. Additionally, factors such as maternal age, province, and access to healthcare significantly influence child health outcomes, with younger mothers (under 20) and those with less education contributing to higher morbidity rates.

Furthermore, the study identifies significant disparities in postnatal care and treatment for childhood illnesses among unwanted children. For instance, children born to non-educated mothers or those from poorer families tend to receive less medical attention, particularly for conditions like diarrhea, fever, and cough. The performance of ML models (RF and NN) also shows the complexity of predicting child morbidity, with RF outperforming NN in several areas, such as fever and ARI prediction. The analysis also underscores the role of socioeconomic factors, such as wealth and maternal education, in influencing the likelihood of receiving treatment. Overall, the findings suggest that improving maternal education, healthcare access, and addressing regional disparities could help reduce the negative health outcomes associated with unwanted pregnancies in Pakistan.

To address the disadvantages of using secondary data, it is important to note that one key limitation is the lack of control over how the data was collected. Researchers using secondary data cannot verify the methods used, which can lead to biases or inconsistencies. Additionally, secondary data may not perfectly align with the research questions, as it was collected for different purposes. There is also the risk of missing or incomplete data, which may affect the analysis and require techniques like imputation. Moreover, secondary data might not have the level of detail needed for specific analysis or may be outdated, which limits its relevance to current situations. Despite these drawbacks, secondary data can still be valuable, but researchers must be cautious and consider these limitations when interpreting the results.

In analyzing childhood morbidity among undesired births, it is essential to consider confounding factors that may influence both birth desirability and health outcomes. Paternal involvement plays a key role in healthcare-seeking behavior; active fathers are more likely to ensure timely medical care, while limited involvement may lead to delays. Maternal mental health, often affected by stress, economic hardship, or unintended pregnancies, can also compromise the quality of care. Additionally, cultural preferences for male children may result in the neglect of undesired female children, leading to disparities in nutrition and healthcare access. Accounting for these factors is crucial for a comprehensive analysis.

The WHO believes that immunization might save 1.5 million deaths in children under the age of five. The poor vaccination rates in the nation are a key contributing factor to the high child mortality figures. Because of widespread disinformation, conspiracy theories, and poor literacy rates, people are very reluctant, if not hostile, about vaccinations. The Pakistan Electronic Media Regulatory Authority should forbid media outlets from disseminating false narratives and penalize them for doing so, as it has the power to control and suspend media outlets’ licenses. Increased fact-checking will result from this, which may help halt the spread of untrue statements like “ vaccines against malaria are a plot by the west”. Hospitals, doctors, and educational institutions should also contribute to the awareness-building process about the advantages of vaccination. To increase immunization rates and promote routine examinations for babies and newborns, hospitals should provide parents with a discount on their subsequent visits to a pediatrician.

Additionally, doctors should educate their own families on the value of vaccinations. This might have a significant influence since individuals are more inclined to heed the advice of dependable family members. Private schools should revise their admissions procedures to accept only fully immunized youngsters and inform parents of un-vaccinated children. In 2019, countries such as Italy enforced regulations prohibiting children under six months old from attending school, while parents of older children were subject to penalties if their kids did not have the necessary vaccinations. Surrounded by other vaccinated children in nurseries and kindergartens, vaccinated children would encourage more parents to vaccinate their children while also lowering the chance of transmission.

Similarly, local religious leaders and celebrities should support the fight against the stigma associated with vaccinations by promoting on social media the advantages of vaccinations. Social media influencers have large followings and can quickly reach a large number of individuals. Religious leaders have a lot of sway in rural places and might persuade more families to vaccinate their kids. These initiatives are required because, despite the fact that important vaccinations are provided free of charge, Pakistan has the third-highest percentage of un-vaccinated children and severely low childhood immunization rates.

## Conclusion

In conclusion, this study focused on the health outcomes and treatment of unwanted children in Pakistan, examining various factors like maternal age, education, socioeconomic status, and place of residence. The findings show that 16.52% of adult mothers (aged 15–49) have an excess of boys, while 34.22% report an excess of girls, and 33.92% experience parity, all contributing to unwanted pregnancies. The analysis found that these children are significantly more likely to suffer from various health conditions. Specifically, 11.21% of unwanted children had diarrhea, 19.53% had a fever, and 24.18% exhibited signs of cough in the past two weeks. Furthermore, 10.27% of boys and 18.55% of girls faced SRB, while 10.44% of children suffered from ARI. The findings revealed that the overall percentage of the undesired child was 8%, 4%, 15%, and 27% for boys, girls, parity, and dual excess, respectively. This data highlights the critical health issues faced by children born from unintended pregnancies, with morbidity rates higher for children born to mothers who are less educated or come from poorer backgrounds.

Moreover, the study highlighted the disparity in healthcare access and treatment for these children. Only 28% of unwanted children received postnatal care within two days of birth, and 46% received treatment for diarrhea in the past six months, significantly lower than the care received by children from more desired pregnancies. ML models, such as RF and NN, were used to predict child morbidity and treatment outcomes, showing that RF was more accurate in identifying predictors. RF achieved 96.27% accuracy in predicting fever cases and 79.2% accuracy for predicting SRB, while NN showed a lower accuracy of 64.5%. The study also highlighted the socio-economic disparities, with 45% of the child morbidity attributed to poor families and 35% to wealthier families, demonstrating how economic status influences health outcomes for unwanted children. These findings emphasize the need for targeted interventions to improve maternal education, healthcare access, and address regional disparities in Pakistan to mitigate the impact of unwanted pregnancies on child health.

The government must strengthen FP initiatives to reduce childhood mortality and morbidity associated with unwanted pregnancies. Promoting the use of modern contraceptives can help prevent unintended births and support progress toward sustainable development goal, which aims to reduce infant and under-five mortality. A key driver of unwanted childbirth is the desire for a specific sex composition, particularly a preference for sons, leading many women to experience repeated pregnancies. It is essential to challenge gender biases and emphasize that daughters, as per Islamic values, are blessings from the God. Addressing gender preference through awareness campaigns is crucial. Additionally, improving maternal and child healthcare services is vital to reduce risks such as low birth weight, abnormal child growth, and unequal treatment. Policymakers must advocate for a clear message that values all children equally and actively works to eliminate son preference and prevent unwanted pregnancies.

## Supporting information

S1 Appendix Supplementary Material. This file contain description of the response and explanatory variables of the study.
(ZIP)
